# Exogenous Hemin alleviates NaCl stress by promoting photosynthesis and carbon metabolism in rice seedlings

**DOI:** 10.1038/s41598-023-30619-7

**Published:** 2023-03-01

**Authors:** Fengyan Meng, Naijie Feng, Dianfeng Zheng, Meiling Liu, Rongjun Zhang, Xixin Huang, Anqi Huang, Ziming Chen

**Affiliations:** 1 College of Coastal Agricultural Sciences, Guangdong Ocean University, Zhanjiang, 524008 China; 2 National Saline-tolerant Rice Technology Innovation Center, South China, Zhanjiang, 524008 China; 3 Shenzhen Institute of Guangdong Ocean University, Shenzhen, 518108 China

**Keywords:** Plant physiology, Plant stress responses

## Abstract

It is widely known that salt stress restricts rice growth and productivity severely. However, little information is available regarding the stage of rice seedlings subjected to the Heme oxygenase 1 (HO-1) inducer, Hemin. This study aimed to investigate the effects of salt stress on two rice varieties (Huanghuazhan and Xiangliangyou 900) and the effect of Hemin in promoting photosynthesis, carbohydrate metabolism, and key enzymes under salt-stress conditions. At the stage of three leaves and one heart, Huanghuazhan (HHZ) and Xiangliangyou 900 (XLY900) were sprayed with 5 μmol·L^−1^ Hemin and then subjected to 50 mM NaCl stress. The results showed that NaCl stress decreased the contents of chlorophyll a, chlorophyll b, and carotenoids. Furthermore, the net photosynthetic rate (*P*_n_) decreased remarkably and the starch content was also lowered. However, NaCl treatment enhanced the concentration of sucrose and soluble sugar, simultaneously enhancing the sucrose metabolism. Nevertheless, the foliar spraying of exogenous Hemin mediated the increase in fructose and starch content, along with the activities of key enzymes’ soluble acid invertase (SAInv), basic/neutral invertase (A/N-Inv), and sucrose synthase (SS) in rice leaves under NaCl stress. The sucrose phosphate synthase (SPS) in leaves decreased significantly, and the fructose accumulation in leaves increased. Hemin also mediated the increase of starch content and the α-amylase, total amylase, and starch phosphorylase (SP) activities under NaCl stress. Under stress conditions, the application of the Heme oxygenase 1 (HO-1) inhibitor, ZnPP failed to alleviate the damage to rice seedlings by NaCl stress. The ZnPP treatment showed similar tendency to the NaCl treatment on pigment content, gas exchange parameters and carbon metabolism related products and enzymes. However, ZnPP decreased carotenoids, fructose, starch content and enzyme activities related to starch metabolism. The regulation effect of Hemin on HuangHuaZhan was better than XiangLiangYou 900. These results indicate that Hemin improved the effects of salt stress on the photosynthesis and physiological characteristics of rice leaves as a result of enhanced carbohydrate metabolism. Thus, Hemin could alleviate the damage caused by salt stress to a certain extent.

## Introduction

Soil salinization is one of the most severe worldwide agricultural problems. Increasingly saline soil is caused by a combination of factors, including unreasonable irrigation, poor drainage, over-exploitation of groundwater, and seawater back-up^1^. According to incomplete statistics, the global saline soils area accounts for 8.7% of the earth’s area, meaning that soil salinity already covers 20% of the total cultivated land and 33% of the irrigated agricultural land worldwide^2^. In China, saline land covers more than 100 million hectares, of which more than 80% is undeveloped^3^. Salt stress is the most widespread and dominant abiotic stress globally, leading not only to wastage of land resources but also has negative impacts on normal crop growth and development processes. It is estimated that salt stress reduces global crop yields by about 20%, resulting in at least $12 billion annually^4^.

Photosynthesis converts solar energy into chemical energy, with sucrose and starch being the main products^5^. The balance between starch storage, reactivation, sucrose synthesis, and export to tissue libraries needs to be maintained in the plant. Among sucrose metabolizing enzymes, SPS catalyses the conversion of UDP-glucose (UDPG) and fructose 6-phosphate (F-6-P) to sucrose. SS and Inv are involved in the cleavage of sucrose. SS is a reversible enzyme that cleaves sucrose to fructose and UDPG. Inv catalyses the irreversible hydrolysis of sucrose to glucose and fructose. The hexose produced by decomposition is used to meet plant growth requirements through other pathways, such as glycolysis^6,7^. The key enzymes involved in starch metabolism are SP and α-amylase. SP degrades starch to glucose 1-phosphate (G-1-P), and also catalyses the production of starch^8^. α-amylase breaks the alpha-1,4-glycosidic bond to produce sugars^9^.

Salt stress increases the soil osmotic pressure, making it difficult for plants to absorb water and nutrients^10,11^. Excessive ions induce stomatal closure to reduce water loss through transpiration and reduce the intercellular carbon dioxide concentration, resulting in a decrease in the net photosynthetic rate^12,13^. Qian et al.^14^ reported that salt stress decreased the values of *P*_n_, *G*_s_, *C*_i_, and *T*_r_. Additionally, salt stress disrupts the chloroplast structure^15^, decreases the content of various chlorophylls in leaves^16^, and destroys the photosynthetic membrane system, thus leading to a disruption of the Calvin cycle and decreases in carbon assimilation^17–19^. In stressful environments, plants generally obtain energy and carbon by breaking down starch. The sugars produced by starch degradation can act as osmoregulatory substances to improve the ability of cells to absorb water^20^. Research showed that under salt stress, the content of starch and soluble sugars dropped, and the content of fructose and sucrose increased, while the activity of enzymes related to sugar metabolism changed in castor cotyledons^21^.

Salt-tolerant plants and halophytes are mainly foraging vegetation, and are not valuable as traditional cash crops^22,23^. Therefore, cultivation of traditional cash crops on saline land increases food production and protects national food security to a certain extent. Rice (*Oryza sativa L.*) is an essential food crop that is vital to the food security of most countries. Rice can be exposed to salt stress at all stages, and at about the 3-leaf stage, it is considered to be particularly susceptible to salt stress^24,25^. Hence, it is essential to improve the salt tolerance of rice at the seedling stage for maintaining normal growth and development and improving yield at the later stage. Previous researchers have shown that the use of plant growth regulators is an effective measure to enhance the salt tolerance of crops^26,27^.

Hemin is a naturally derived chloride of heme from animal blood and can be used as an effective promoter of heme oxygenase-1 (HO-1), which is broken down by HO-1 to produce carbon monoxide (CO), biliverdin (BV), and ferrous ions (Fe^2+^)^28,29^. Its structure is a Fe^3+^ complexed protoporphyrin, which is similar to chlorophyll^30^. Hemin boosts the growth of the lateral roots of tomato^31^, promotes the growth of cucumber roots^32^, and increases the anthocyanin content of radish^33^. Research has proven that Hemin as a highly effective anti-stress agent is effective in alleviating abiotic stresses, such as heavy metal stress^34^, low-temperature stress^30^, and salt stress^35^. The spraying of Hemin on maize increased the chlorophyll content and net photosynthetic rate, delayed leaf senescence, and improved the accumulation and distribution of photosynthetic products. This further encouraged the activity and gene expression of key enzymes for starch synthesis, strengthened cadmium resistance, and raised maize yields^36^. Previous study showed that exogenous Hemin mitigated the oxidative damage caused by cadmium stress in cabbage by increasing the chlorophyll content, enhancing photosynthesis, activating the antioxidant system, and up-regulating the expression of transporter protein genes^37^.

In plants, carbon metabolism is sensitive to environmental stresses and it is closely associated with other metabolic pathways. It not only provides carbon and energy, but also produces substances with signal molecular functions, such as sucrose and glucose. At present, the effects of Hemin on carbon metabolism in rice seedlings under NaCl stress are understood much less, and the specific regulatory mechanisms are still unknown. The experiment in this research was conducted on rice production. Hemin was sprayed first, followed by inducing NaCl stress. Rice seedlings were treated with a regulator and transplanted into saline soils. This experiment used conventional rice Huanghuazhan (HHZ) and hybrid rice Xiangliangyou 900 (XLY900) as test materials and investigated the mechanism of the Hemin regulation of chlorophyll content, photosynthesis, carbon metabolite content, and related enzyme (SS, SPS, SP, etc.) activities in rice leaves under NaCl stress. This study contributes to our understanding of the regulatory role of Hemin in carbon metabolism and provides new insights into the regulation of rice salt tolerance by Hemin.

## Results

### Effect of Hemin on seedling growth of rice under salt stress

**Figure 1:**
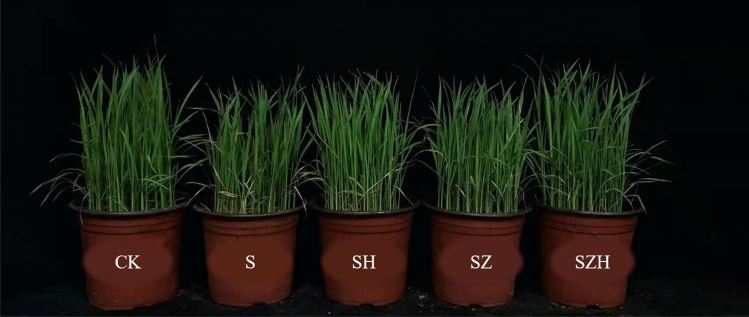
Effects of Hemin on the growth of rice seedlings under NaCl stress (5 d) in HHZ. CK: 0 mM NaCl + 0 μmol·L^−1^Hemin, S: 50 mM NaCl + 0 μmol·L^−1^Hemin, SH: 50 mM NaCl + 5 μmol·L^−1^Hemin, SZ: 50 mM NaCl + 25 μmol·L^−1^ZnPP, SZH:50 mM NaCl + 5 μmol·L^−1^Hemin + 25 μmol·L^−1^ZnPP.

**Figure 2:**
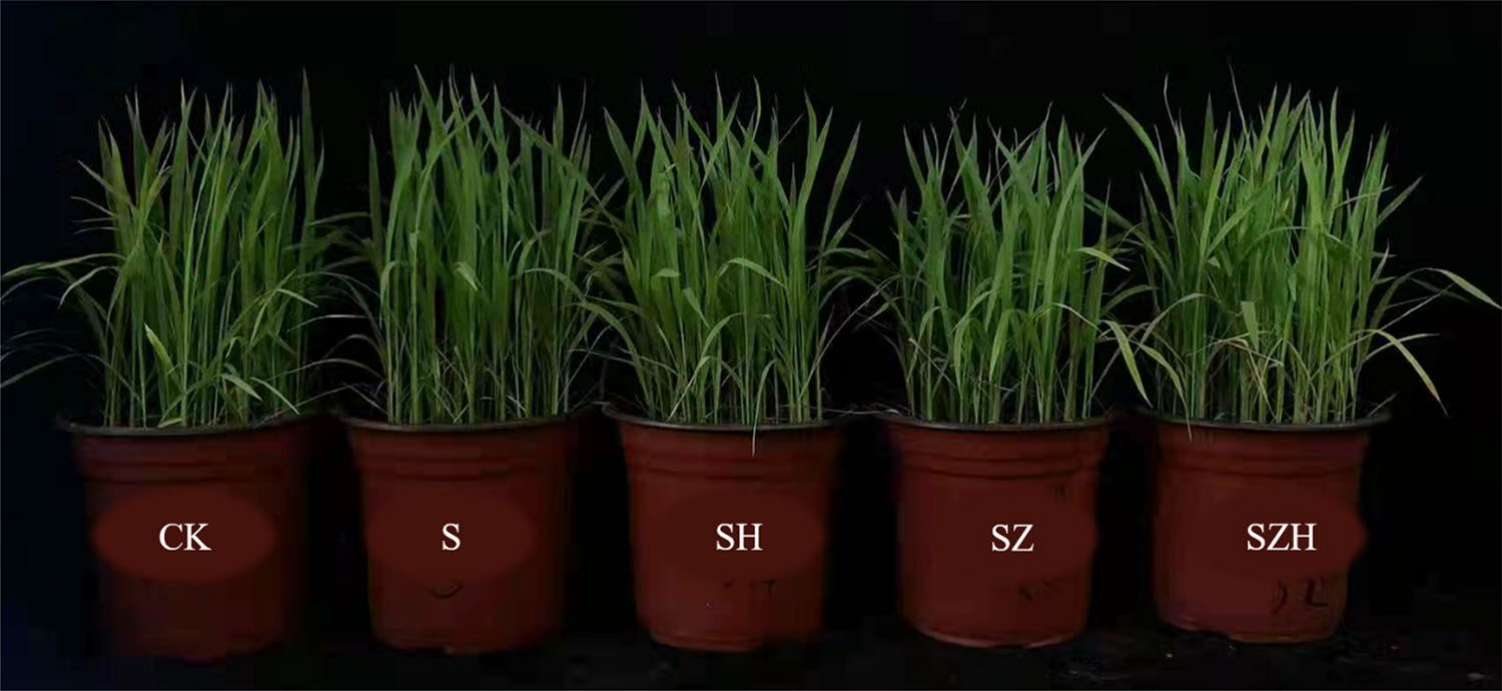
Effects of Hemin on the growth of rice seedlings under NaCl stress (5 d) in XLY900. CK: 0 mM NaCl + 0 μmol·L^−1^Hemin, S: 50 mM NaCl + 0 μmol·L^−1^Hemin, SH: 50 mM NaCl + 5 μmol·L^−1^Hemin, SZ: 50 mM NaCl + 25 μmol·L^−1^ZnPP, SZH:50 mM NaCl + 5 μmol·L^−1^Hemin + 25 μmol·L^−1^ZnPP.

**Figure 3:**
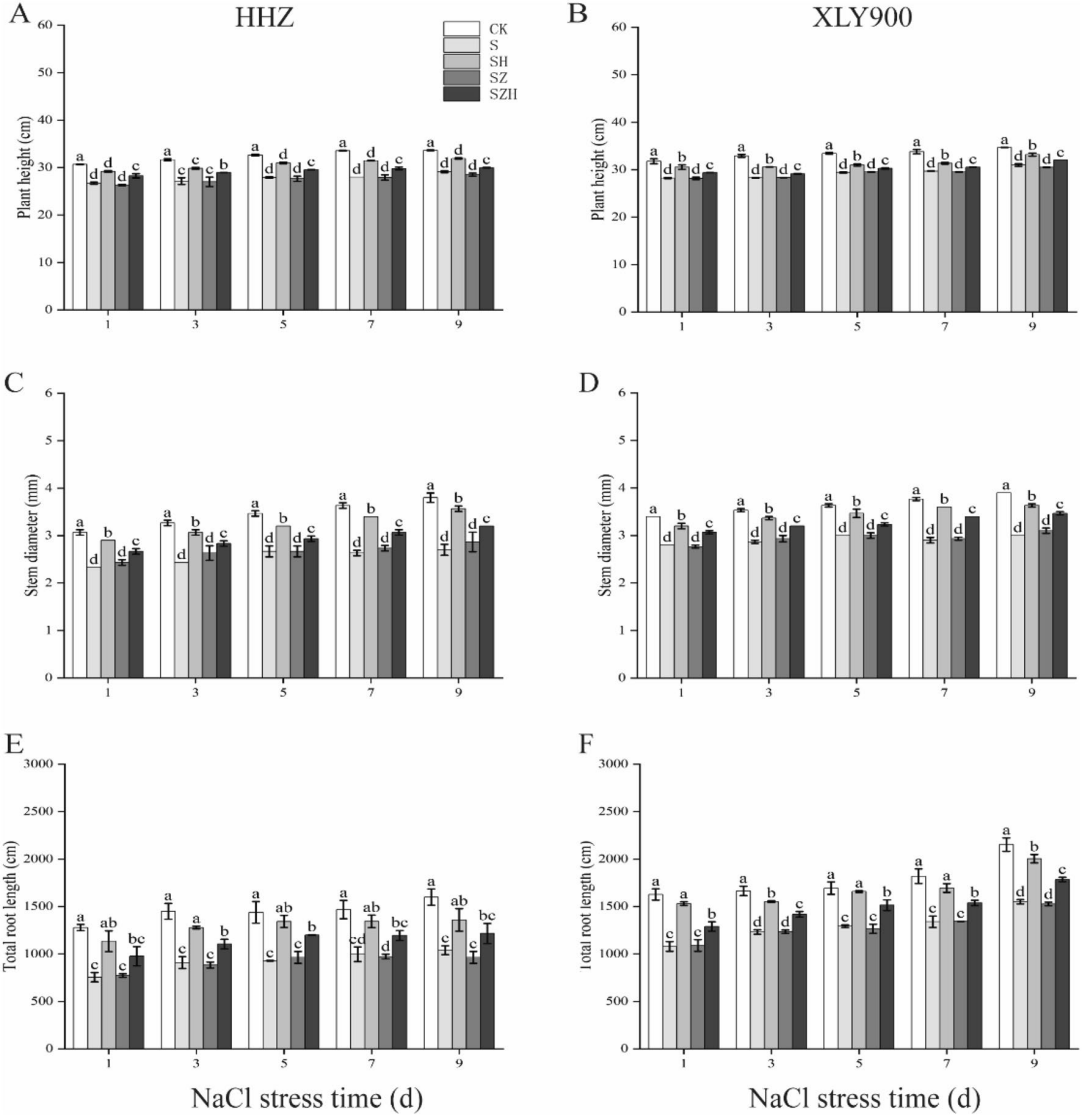
Effect of Hemin on seedling growth of rice under salt stress. plant height in HHZ (**A**) and XLY900 (**B**); stem base width in HHZ (**C**) and XLY900 (**D**); and total root length in HHZ (**E**) and XLY900 (**F**). CK: 0 mM NaCl + 0 μmol·L^−1^Hemin, S: 50 mM NaCl + 0 μmol·L^−1^Hemin, SH: 50 mM NaCl + 5 μmol·L^−1^Hemin, SZ: 50 mM NaCl + 25 μmol·L^−1^ZnPP, SZH:50 mM NaCl + 5 μmol·L^−1^Hemin + 25 μmol·L^−1^ZnPP. Values are the mean ± SD of three replicate samples. Different letters in the data column indicate significant differences (p < 0.05) according to Duncan’s test.

Under NaCl stress, rice growth was obviously inhibited (Figs. 1, 2), as shown that morphological indicators such as plant height, stem base width and total root length were significantly decreased (Fig. 3A–F). In comparison to CK, the plant height, stem base width and total root length of HHZ reduced significantly, by 13–17%, 23–29% and 32–41%, respectively, under S treatment from day 1 through 9. In XLY900, they significantly decreased, by 11–14%, 17–23% and 24–34%, respectively. NaCl stress was more effective in inhibiting the growth of HHZ than XLY900. The application of exogenous hemin alleviated the inhibitory effect of NaCl stress on the growth of rice seedlings. In comparison to the S treatment, the plant height, stem base width and total root length of HHZ with SH treatment were significantly higher, by 9–12% 14–24% and 31–50%, from day 1 through 9, respectively; the plant height, stem base width and total root length of XXL900 with SH treatment were significantly enhanced, by 5–8%, 20–32% and 26–42%, respectively. In comparison to S treatment, the ZnPP treatment of both rice varieties failed to promote the growth of rice seedlings. The addition of Hemin reversed the ZnPP-induced inhibition effect. In comparison to the SZ treatment, the plant height, stem base width and total root length of HHZ with SZH treatment were higher, by 5–7%, 8–12% and 23–26%, from day 1 through 9, respectively; the plant height, stem base width and total root length of XXL900 with SZH treatment were enhanced, by 3–4%, 8–16% and 14–20%, respectively.

### Effect of Hemin on seedling biomass of rice under salt stress

**Figure 4:**
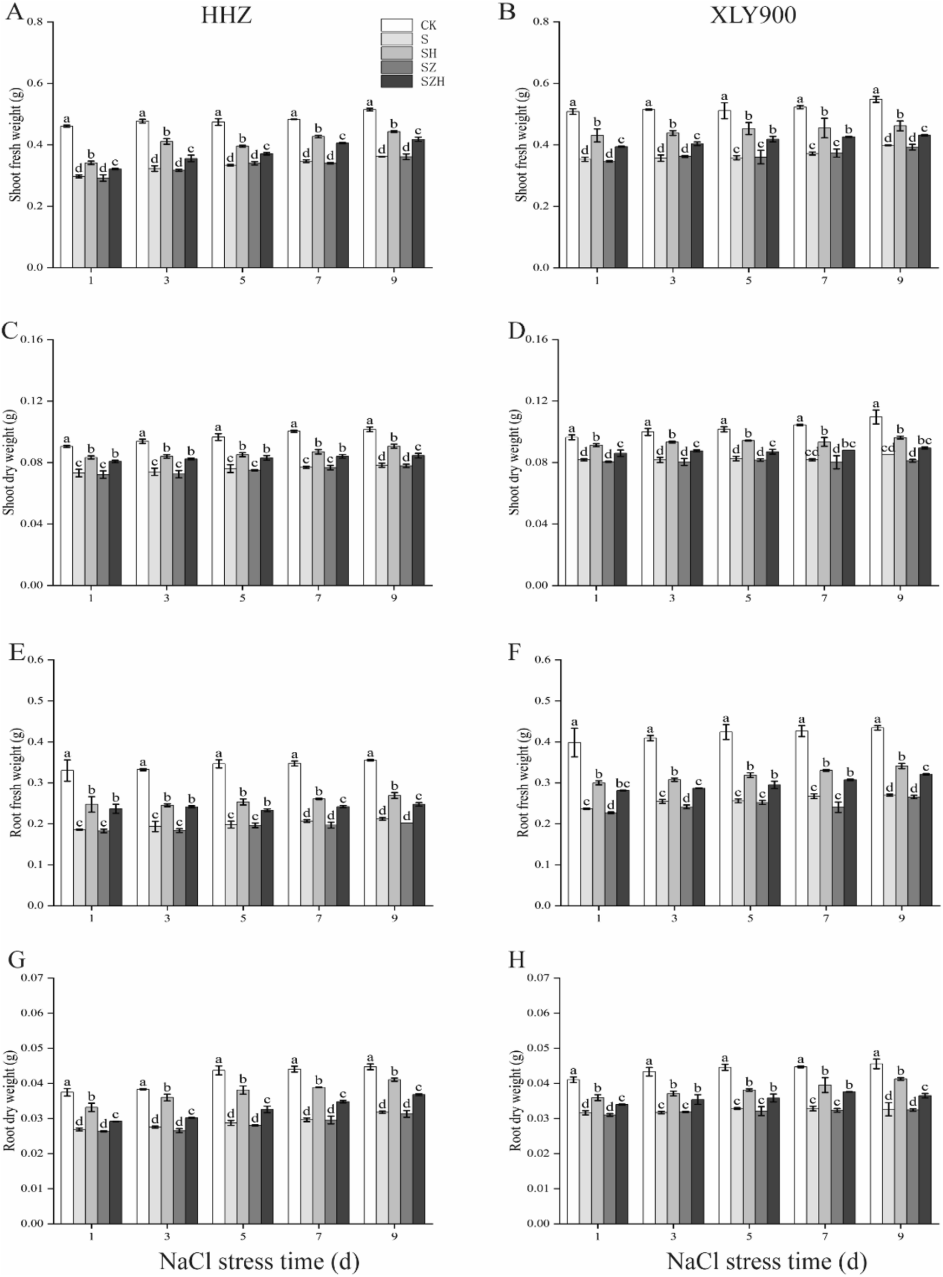
Effect of Hemin on seedling biomass of rice under salt stress. Shoot fresh weight in HHZ (**A**) and XLY900 (**B**); shoot dry weight in HHZ (**C**) and XLY900 (**D**); root fresh weight in HHZ (**E**) and XLY900 (**F**); root dry weight in HHZ (**G**) and XLY900 (**H**). CK: 0 mM NaCl + 0 μmol·L^−1^Hemin, S: 50 mM NaCl + 0 μmol·L^−1^Hemin, SH: 50 mM NaCl + 5 μmol·L^−1^Hemin, SZ: 50 mM NaCl + 25 μmol·L^−1^ZnPP, SZH:50 mM NaCl + 5 μmol·L^−1^Hemin + 25 μmol·L^−1^ZnPP. Values are the mean ± SD of three replicate samples. Different letters in the data column indicate significant differences (p < 0.05) according to Duncan’s test.

The shoot fresh and dry weights of both rice varieties were significantly decreased under NaCl treatment (Fig. 4A–D). In comparison to CK, the shoot fresh weight and dry weight of HHZ decreased significantly, by 28–35% and 19–23%, respectively, under S treatment from day 1 through 9. In XLY900, they significantly decreased, by 27–31% and 15–22%, respectively. The application of exogenous Hemin improved biomass accumulation in HHZ and XLY900. In comparison to the S treatment, the shoot fresh weight and dry weight of HHZ with SH treatment were significantly higher, by 15–27% and 12–16%, from day 1 through 9, respectively; the shoot fresh weight and dry weight of XXL900 with SH treatment were significantly enhanced, by 16–27% and 12–14%, respectively. In comparison to S treatment, the ZnPP treatment of both rice varieties did not promote biomass accumulation. The addition of Hemin reversed the ZnPP-induced inhibition effect. In comparison to the SZ treatment, the shoot fresh weight and dry weight of HHZ with SZH treatment were higher, by 9–19% and 9–14%, from day 1 through 9, respectively; the shoot fresh weight and dry weight of XXL900 with SZH treatment were enhanced, by 10–16% and 7–10%, respectively.

The root fresh and dry weights of both rice varieties were significantly declined under NaCl treatment (Fig. 4E–H). In comparison to CK, the root fresh weight and dry weight of HHZ decreased significantly, by 40–44% and 28–34%, respectively, under S treatment from day 1 through 9. In XLY900, they significantly decreased, by 37–41% and 23–28%, respectively. The application of exogenous Hemin improved root fresh and dry weights in HHZ and XLY900. In comparison to the S treatment, the root fresh weight and dry weight of HHZ with SH treatment were significantly higher, by 26–33% and 23–33%, from day 1 through 9, respectively; the shoot fresh weight and dry weight of XXL900 with SH treatment were significantly enhanced, by 21–27% and 14–26%, respectively. In comparison to S treatment, the ZnPP treatment of both rice varieties did not produce any boosting effects. The addition of Hemin reversed the ZnPP-induced inhibition effect. In comparison to the SZ treatment, the shoot fresh weight and dry weight of HHZ with SZH treatment were higher, by 19–32% and 11–18%, from day 1 through 9, respectively; the shoot fresh weight and dry weight of XXL900 with SZH treatment were enhanced, by 17–28% and 10–16%, respectively.

### Effect of Hemin on the photosynthetic pigment content in rice under salt stress

**Figure 5:**
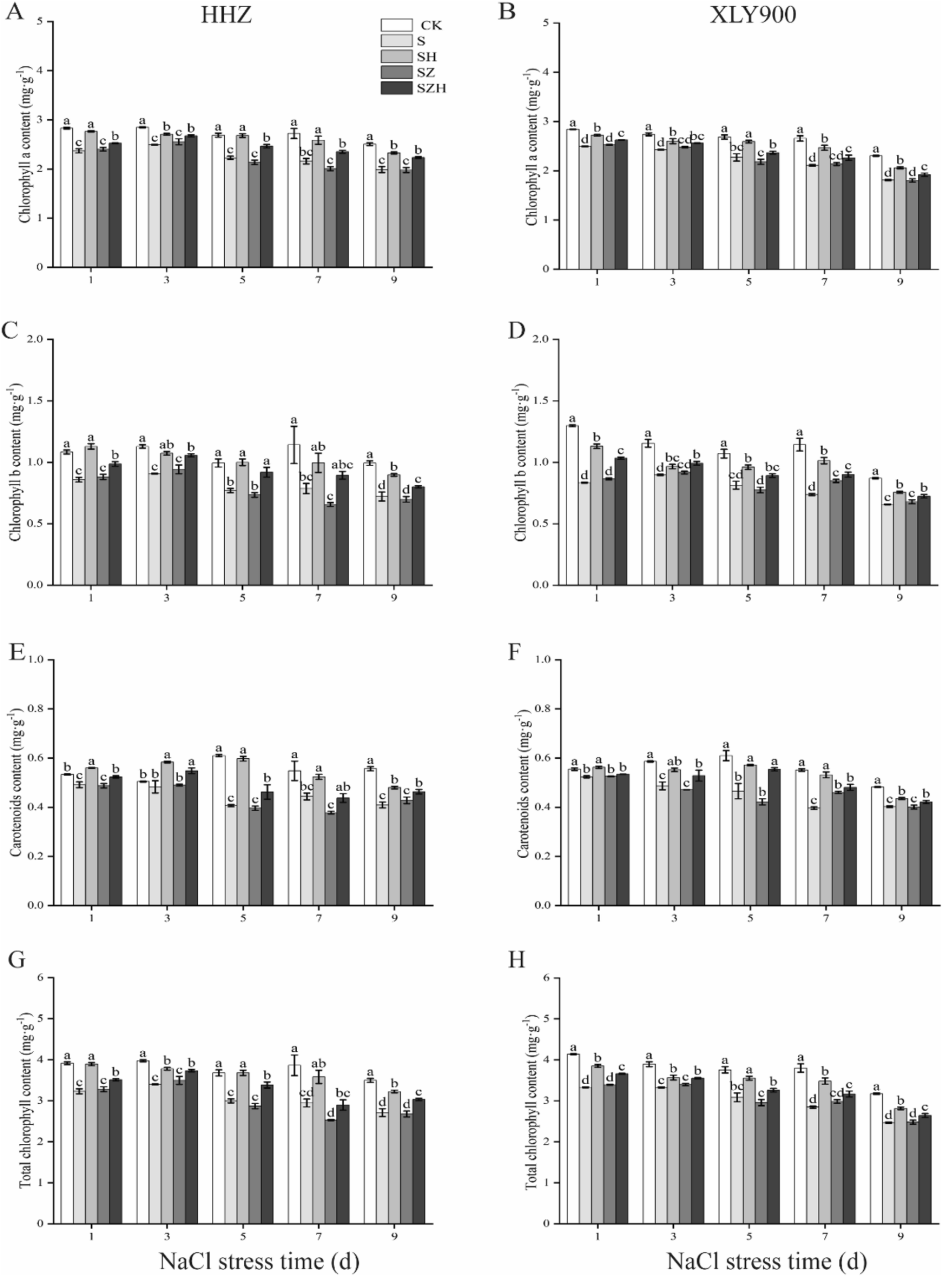
Effect of Hemin on photosynthetic pigment content in rice under salt stress. Chlorophyll a content in HHZ (**A**) and XLY900 (**B**); Chlorophyll b content in HHZ (**C**) and XLY900 (**D**); Carotenoids content in HHZ (**E**) and XLY900 (**F**); Total chlorophyll in HHZ (**G**) and XLY900 (**H**). CK: 0 mM NaCl + 0 μmol·L^−1^Hemin, S: 50 mM NaCl + 0 μmol·L^−1^Hemin, SH: 50 mM NaCl + 5 μmol·L^−1^Hemin, SZ: 50 mM NaCl + 25 μmol·L^−1^ZnPP, SZH:50 mM NaCl + 5 μmol·L^−1^Hemin + 25 μmol·L^−1^ZnPP. Values are the mean ± SD of three replicate samples. Different letters in the data column indicate significant differences (p < 0.05) according to Duncan’s test.

Under NaCl stress, the synthesis of chlorophyll in rice leaves was stunted (Fig. 5A–H). In comparison to CK, the chlorophyll a, chlorophyll b, carotenoids, and total chlorophyll content of HHZ under NaCl stress reduced by 12–21%, 20–31%, 4–33%, and 14–24% from day 1 through 9, respectively; for XLY900, the abovementioned indicators significantly decreased by 11–21%, 22–36%, 6–28%, and 15–25%. In comparison to S treatment, the chlorophyll a, chlorophyll b, carotenoids, and total chlorophyll content of HHZ with SH treatment notably increased, by 9–20%, 18–31%, 14–47%, and 11–23% from day 1 through 9, respectively. For XLY900, the parameters increased by 7–17%, 8–37%, 7–34%, and 7–22%. The data showed that Hemin had stronger mitigation effect on the decrease in chlorophyll content in rice under NaCl stress. However, ZnPP treatment could not alleviate the inhibitory effect of NaCl stress on the chlorophyll content in both rice seedlings. On day 7, in comparison to S treatment, the chlorophyll a, chlorophyll b, carotenoids, and total chlorophyll content of HHZ with SZ treatment decreased approximately 7%, 17%, 15% and 14% in respectively. On day 5, in comparison to S, the chlorophyll a, chlorophyll b, carotenoids, and total chlorophyll content of XLY900 with SZ treatment decreased approximately 4%, 5%, 9% and 4% in respectively. This inhibition could be reversed partially by combining Hemin with ZnPP. On day 7, in comparison to SZ treatment, the chlorophyll a, chlorophyll b, carotenoids, and total chlorophyll content of HHZ with SZH treatment increased approximately 17%, 36%, 16% and 15% in respectively. On day 5, in comparison to SZ treatment, the chlorophyll a, chlorophyll b, carotenoids, and total chlorophyll content of XLY900 with SZH treatment increased approximately 9%, 15%, 31% and 10% in respectively.

### Effect of Hemin on the gas exchange parameters in rice under salt stress

**Figure 6:**
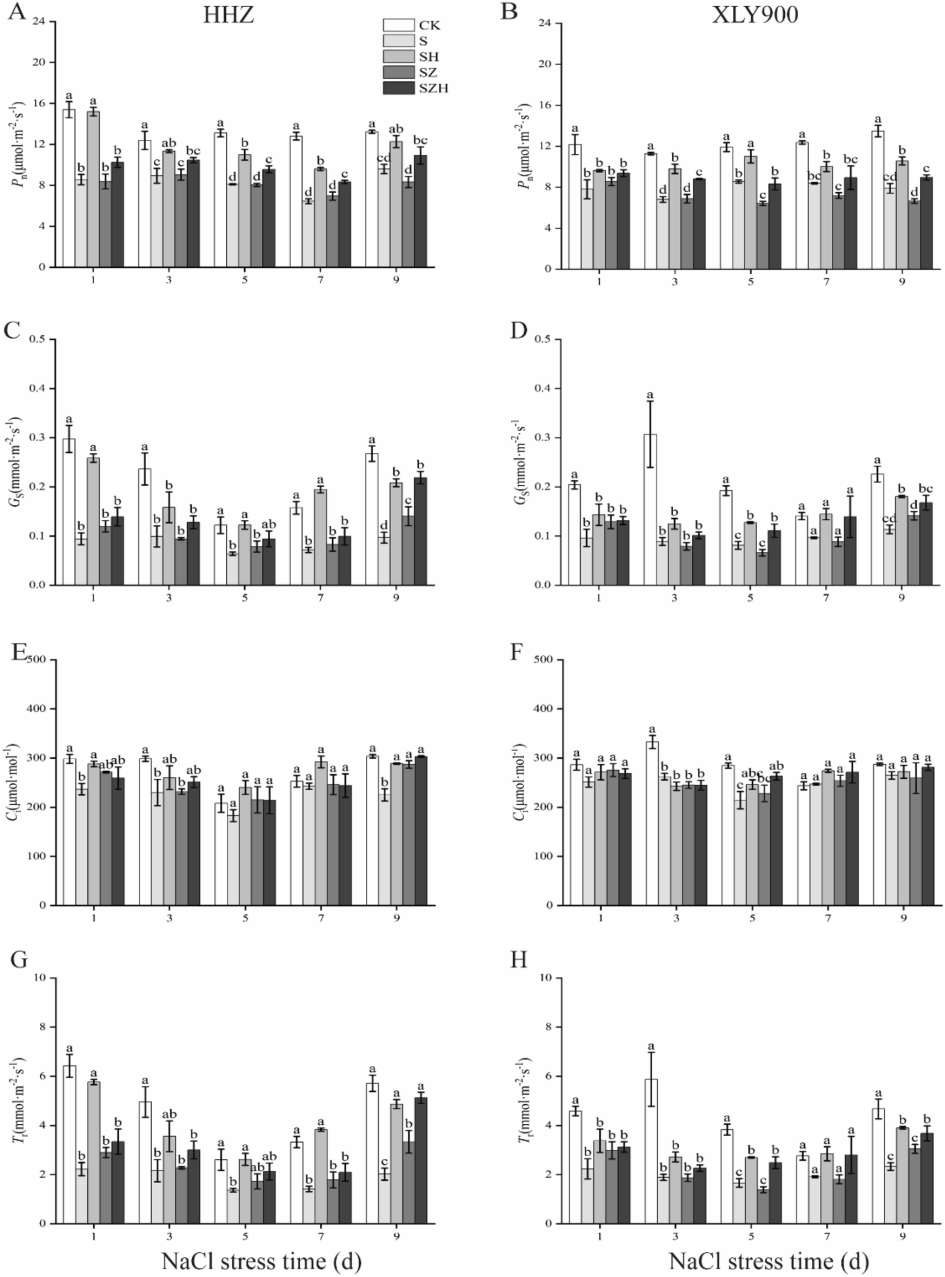
Effect of Hemin on gas exchange parameters in rice under salt stress. *P*_n_ in HHZ (**A**) and XLY900 (**B**); *G*_s_ in HHZ (**C**) and XLY900 (**D**); *C*_i_ in HHZ (**E**) and XLY900 (**F**); and *T*_r_ in HHZ (**G**) and XLY900 (**H**). CK: 0 mM NaCl + 0 μmol·L^−1^Hemin, S: 50 mM NaCl + 0 μmol·L^−1^Hemin, SH: 50 mM NaCl + 5 μmol·L^−1^Hemin, SZ: 50 mM NaCl + 25 μmol·L^−1^ZnPP, SZH:50 mM NaCl + 5 μmol·L^−1^Hemin + 25 μmol·L^−1^ZnPP. Values are the mean ± SD of three replicate samples. Different letters in the data column indicate significant differences (p < 0.05) according to Duncan’s test.

NaCl stress decreased the photosynthesis rate (*P*_n_), stomatal conductance (*G*_s_), internal CO_2_ concentration (*C*_i_), and transpiration rate (*T*_r_) values in HHZ and XLY900 (Fig. 6A–H). Except for *C*_i_, *P*_n_, *G*_s_, and *T*_r_ reached significant difference levels at all five time points. In comparison to CK, the *P*_n_, *G*_s_, and *T*_r_ values of HHZ with S treatment were markedly decreased, by 27–50%, 48–68%, and 48–65% from day 1 through 9, respectively. The *P*_n_, *G*_s_, and *T*_r_ of XLY900 with S treatment were observably reduced, by 28–42%, 31–71%, and 31–68%, respectively. The application of exogenous Hemin mitigated the inhibitory effect of NaCl stress on photosynthesis in two rice cultivars (Fig. 6A–H). On day 3, in comparison to S treatment, the *P*_n_, *G*_s_, *C*_i_, and *T*_r_ in HHZ with SH treatment were dramatically increased, by about 27%, 60%, 13%, and 65%, respectively; in contrast, *P*_n_, *G*_s_, and *T*_r_ were increased by about 44%, 41%, and 44%, respectively, *C*_i_ was reduced by 7% in XLY900 with SH treatment. ZnPP, an inhibitor of HO-1, exacerbated the effects of NaCl stress on *P*_n_, *G*_s_, *C*_i_, and *T*_r_. The combination with Hemin relieved the adverse effects of ZnPP and improved some photosynthetic parameters. On day 5, day 7, and day 9, compared with SZ treatment, the *P*_n_ of HHZ with SZH treatment was increased, by 19%, 20%, and 31%, respectively; in XLY900 with SZH treatment, it was dramatically enhanced, by 30%, 24% and 35%, respectively. On day 9, in comparison to SZ treatments, two varieties of SZH treatments reached a significant level of difference in *G*_*s*_. *G*_*s*_ in HHZ and XLY900 with SZH treatment significantly increased by 56% and 19%, respectively. In comparison to SZ treatment, there was a slight difference in *C*_*i*_ of HHZ and XLY900 with SZH treatment during treatment time course. For example, on day 9, the *C*_*i*_ of HHZ and XLY900 with SZH treatment was increased by 6% and 9%, respectively. On day 9, in comparison to SZ treatments, the *T*_*r*_ of HHZ and XLY900 with SZH treatment was increased by 53% and 21%, respectively.

### Effect of Hemin on the carbohydrate content in rice under salt stress

**Figure 7:**
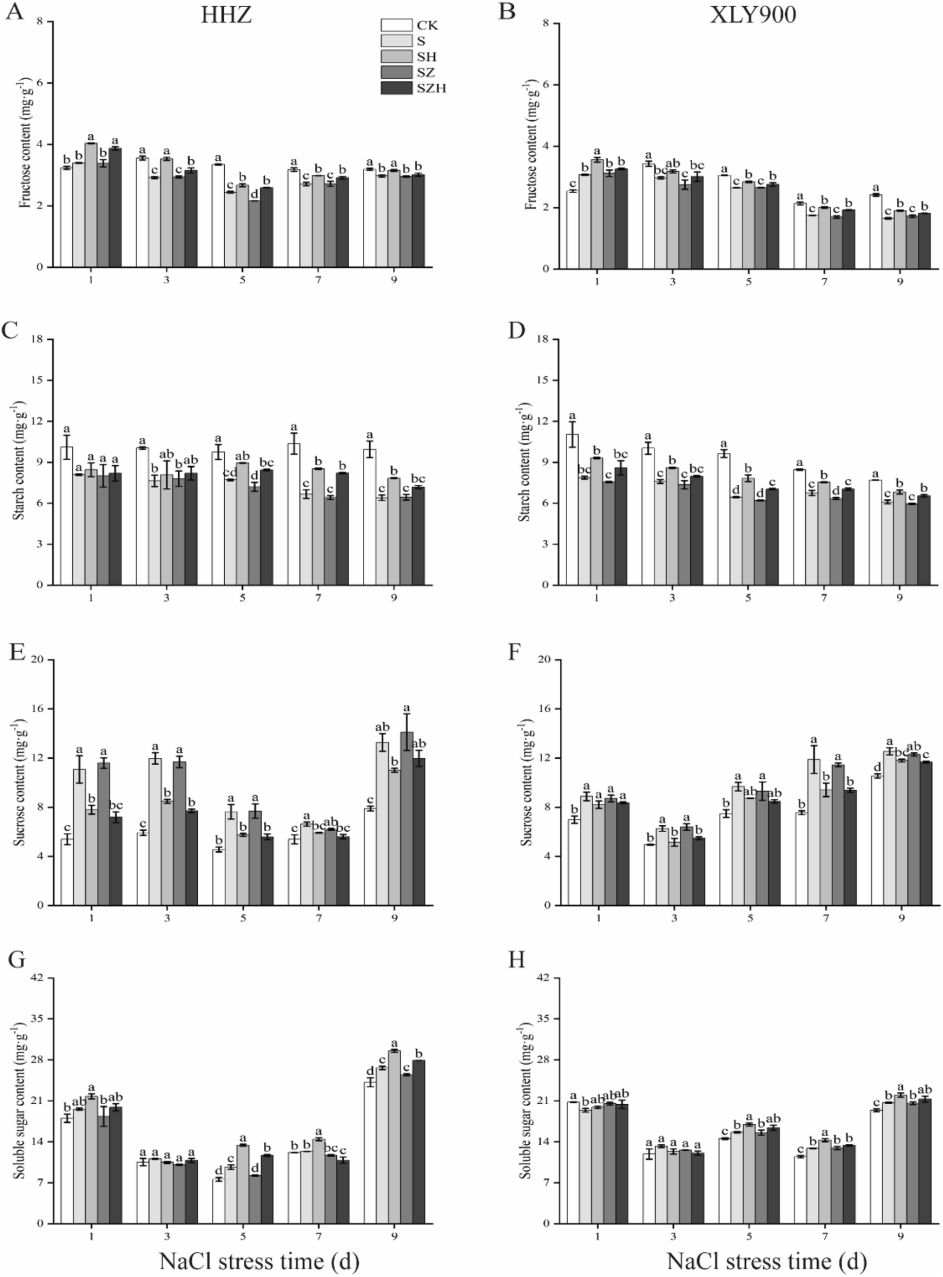
Effect of Hemin on the carbohydrate content in rice under salt stress. Fructose content in HHZ (**A**) and XLY900 (**B**); starch content in HHZ (**C**) and XLY900 (**D**); sucrose content in HHZ (**E**) and XLY900 (**F**); and soluble sugar content in HHZ (**G**) and XLY900 (**H**). CK: 0 mM NaCl + 0 μmol·L^−1^Hemin, S: 50 mM NaCl + 0 μmol·L^−1^Hemin, SH: 50 mM NaCl + 5 μmol·L^−1^Hemin, SZ: 50 mM NaCl + 25 μmol·L^−1^ZnPP, SZH:50 mM NaCl + 5 μmol·L^−1^Hemin + 25 μmol·L^−1^ZnPP. Values are the mean ± SD of three replicate samples. Different letters in the data column indicate significant differences (p < 0.05) according to Duncan’s test.

The accumulation of sugars followed different trends in rice leaves experiencing NaCl stress. Firstly, under NaCl stress, the fructose and starch contents of HHZ and XLY900 decreased with increasing stress times (Fig. 7A–D). In comparison to CK, the fructose and starch content of HHZ under NaCl stress reduced by 7–27% and 20–36% from day 3 through 9, respectively; for XLY900, the fructose and starch content decreased by 13–31% and 20–33%, respectively. The Hemin treatment raised the fructose and starch content of the two rice varieties. In comparison to S treatment, the fructose content of HHZ and XLY900 with SH treatment was higher, by 6–21% and 7–16%, respectively from day 1 through 9. In comparison to S treatment, the starch content of HHZ and XLY900 with SH treatment increased, by 4–28% and 12–21%, respectively from day 1 through 9. In contrast, the ZnPP treatment reduced the fructose and starch content in the two rice varieties. The combination with Hemin relieved the adverse effects of ZnPP and fructose, increasing the starch content. For example, on day 5, in comparison to SZ treatment, the fructose and starch contents of the HHZ with SZH treatment significantly increased, by 20% and 17%, and were significantly increased, by 4% and 13% in the XLY 900 with SZH treatment.

Next, it was found that the sucrose content was significantly elevated under NaCl stress (1–9 d) in the S treatments of the two rice cultivars, and the soluble sugar content was dramatically increased in the later days of stress from day 5 through 9 (Fig. 7E–H). The application of exogenous Hemin decreased the sucrose content. In comparison to S treatment, the sucrose content of HHZ and XLY900 with SH treatment decreased by 11–30% and 6–21%, respectively, from day 1 through 9. Meanwhile Hemin increased the soluble sugar content in rice leaves. On day 5, day 7, and day 9, the soluble sugar content of HHZ with SH treatment was 39%, 17%, and 11% higher than those of S treatment, respectively; while for XLY900 SH treatment was 9%, 11%, and 6% higher. The soluble sugar and sucrose contents were slightly lower in the ZnPP treatment than the S treatment in the two rice varieties. This downward trend could be reversed partially through the combination of Hemin with ZnPP. In comparison to SZ treatment, the sucrose content of HHZ and XLY900 with SZH treatment decreased by 10–38% and 4–18%, respectively, from day 1 through 9. On day 5, in comparison to SZ treatment, the soluble sugar content of HHZ with SZH treatment increased by 42%. On day 5, day 7, and day 9, in XLY900 with SZH treatment, the soluble sugar content increased 5%, 4% and 3%, respectively.

### Effect of Hemin on sucrose metabolism related enzymes in rice under salt stress

**Figure 8:**
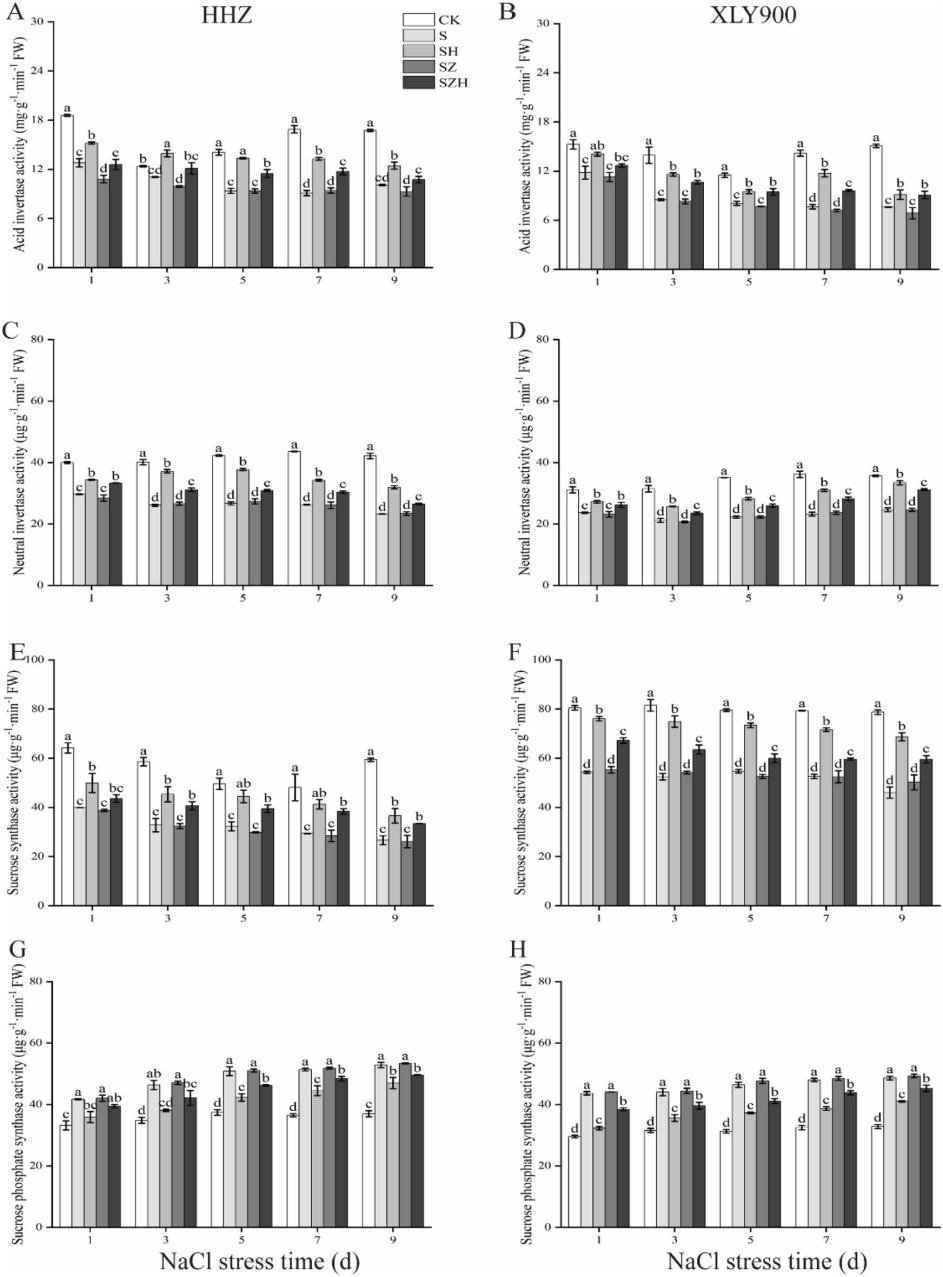
Effect of Hemin on sucrose metabolism related enzymes in rice under salt stress. acid invertase activity in HHZ (**A**) and XLY900 (**B**); neutral invertase activity in HHZ (**C**) and XLY900 (**D**); sucrose synthase activity in HHZ (**E**) and XLY900 (**F**); and sucrose phosphate synthase activity in HHZ (**G**) and XLY900 (**H**). CK: 0 mM NaCl + 0 μmol·L^−1^Hemin, S: 50 mM NaCl + 0 μmol·L^−1^Hemin, SH: 50 mM NaCl + 5 μmol·L^−1^Hemin, SZ: 50 mM NaCl + 25 μmol·L^−1^ZnPP, SZH:50 mM NaCl + 5 μmol·L^−1^Hemin + 25 μmol·L^−1^ZnPP. Values are the mean ± SD of three replicate samples. Different letters in the data column indicate significant differences (p < 0.05) according to Duncan’s test.

The activities of acid invertase (AI), neutral invertase (NI), and sucrose synthase (SS) in leaves followed the same trend with increases in the NaCl stress time (Fig. 8A–F). In comparison to CK, the abovementioned indicators were significantly reduced, by 11–46%, 26–45%, and 35–55% in HHZ with S treatment, and markedly decreased, by 23–50%, 24–36%, and 31–42% in XLY900, respectively. The application of exogenous Hemin increased the activities of AI, NI, and SS. In HHZ with SH treatment, AI, NI, and SS activities were markedly higher than those with S treatment, by 19–46%, 16–42%, and 25–40%, respectively. In XLY900 with SH treatment, they were significantly higher than those with S treatment, by 18–54%, 15–36%, and 34–49%, respectively. The addition of the inhibitor ZnPP reduced these enzymes activities. In comparison to the S treatment, the AI activity of HHZ with SZ treatment was significantly decreased, by 16%, on day 1. ZnPP, in combination with Hemin, increased these enzymes activities in the two types of rice leaves. From day 1 through 9, compared with SZ treatment, the above-mentioned enzyme activities were increased by 16–25%, 13–17%, and 13–35% in HHZ with SZH treatment, were increased by 12–34%, 13–27%, and 14–22% in XLY900 with SZH treatment, respectively.

The activity of sucrose phosphate synthase (SPS) in leaves exhibited a significant increase under NaCl stress (Fig. 8G,H). In comparison to CK, in HHZ and XLY900 with S treatment, the activity of SPS was significantly enhanced, by 26–43% and 40–48%, respectively. The application of exogenous Hemin significantly diminished enzyme activity. From day 1 through 9, in comparison to S treatment, the SPS activity of HHZ and XLY900 SH treatment was effectively degraded, by 11–18% and 16–26%, respectively. However, the addition of the inhibitor ZnPP failed to improve the effect of NaCl stress on SPS activity. Among them, HHZ with SZ treatment had the maximum increase of about 1%, while XLY900 SZ treatment had the largest increase of 3%. In contrast, the inhibition could be reversed partially by the addition of Hemin. During the period of stress, in comparison to SZ treatment, the SPS activity of HHZ and XLY900 with SZH treatment was slightly decreased. From day 1 through 9, in comparison to SZ treatment, the enzyme activity of HHZ and XLY900 with SZH treatment was declined, by 6%–10% and 8%–13%, respectively.

### Effect of Hemin on starch metabolism related enzymes in rice under salt stress

**Figure 9:**
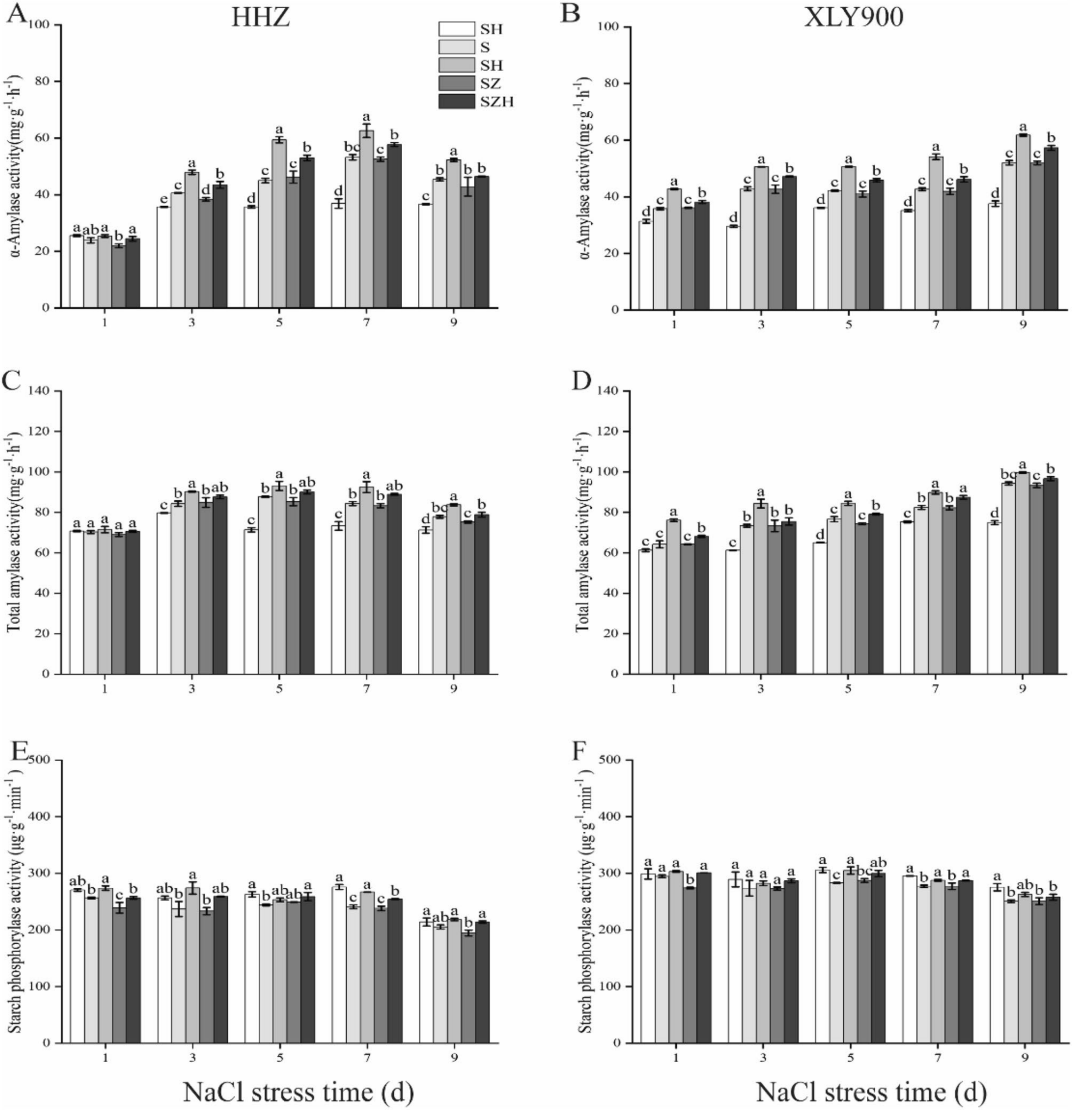
Effect of Hemin on starch metabolism related enzymes in rice under salt stress. α-amylase activity in HHZ (**A**) and XLY900 (**B**); total amylase activity in HHZ (**C**) and XLY900 (**D**) and starch phosphorylase activity in HHZ (**E**) and XLY900 (**F**). CK: 0 mM NaCl + 0 μmol·L^−1^Hemin, S: 50 mM NaCl + 0 μmol·L^−1^Hemin, SH: 50 mM NaCl + 5 μmol·L^−1^Hemin, SZ: 50 mM NaCl + 25 μmol·L^−1^ZnPP, SZH:50 mM NaCl + 5 μmol·L^−1^Hemin + 25 μmol·L^−1^ZnPP. Values are the mean ± SD of three replicate samples. Different letters in the data column indicate significant differences (p < 0.05) according to Duncan’s test.

The activities of α-amylase and total amylase in leaves exhibited increases at higher NaCl stress times (Fig. 9A–D). In comparison to CK, the α-amylase and total amylase activities of HHZ with S treatment were dramatically increased on day 3, 5, 7, and 9. In XLY900 with S treatment, α-amylase was significantly increased from day 1 through 9, while the total amylase was significantly enhanced on day 3, 5, 7, and 9. After spraying with Hemin, the α-amylase and total amylase of both rice varieties were further improved. In comparison to S treatment, the α-amylase and total amylase of HHZ with SH treatment were significantly increased by 15–32% and 6–10% from day 3 through 9, respectively. In XLY900 with SH treatment, the α-amylase and total amylase were increased by 18–27% and 6–19% from day 1 through 9, respectively. The α-amylase and total amylase activities in the SZ treatment of two rice varieties were lower than those in S treatment. On day 3, the α-amylase of HHZ with SZ treatment was significantly reduced, by 5%, in comparison to the S treatment. On day 5, the α-amylase of XLY900 with SZ treatment was reduced, by 3%, in comparison to the S treatment. On day 5, the total amylase of HHZ and XLY900 with SZ treatment was reduced, by 3% and 3%, respectively, in comparison to the S treatment. In contrast, the reduction could be reversed partially by the addition of Hemin. On day 5, they were increased by 15% and 6% in HHZ with SZH treatment, and enhanced by 12% and 6% in XLY900 with SZH treatment, respectively.

Under NaCl stress, the starch phosphorylase activity (SP) of the two rice varieties decreased with increasing stress times (Fig. 9E,F). On day 7, the enzyme activity of HHZ and XLY900 was about 13% and 6% lower than those of CK, respectively. The use of exogenous Hemin significantly improved the enzyme activity. On day 7, in comparison to S treatment, the SP of HHZ and XLY900 with SH treatment were significantly increased, by 11% and 4%, respectively. There were minor differences in the two varieties in SZ treatments when compared with S treatments during treatment time course, expect on day 1, this difference reached a significant level. The SP activity of HHZ and XLY900 with SZ treatment was significantly reduced, by about 7% and 7%, respectively, on day 1. The downward trend could be reversed partially through a combination of Hemin with ZnPP. From day 1 through 9, in comparison to SZ treatment, the SP activity of HHZ and XLY900 with SZH treatment was significantly increased, by 4–11% and 3–10%, respectively.

## Discussion

Excessive salinity adversely affects plant growth by reducing the activity of meristem cells and interfering with normal physiological and biochemical processes^38^. Many studies showed that excessive salinity inhibited new leaf growth and development of root systems and eventually reduced biomass accumulation^39^. This study confirmed that NaCl stress severely restricted the growth and development of rice (Figs. 1, 2 and 3) and significantly reduced the above-ground fresh weight and dry weight of HHZ and XLY900 (Fig. 4). Under NaCl stress, in HHZ, the greatest decrease in shoot fresh weight and dry weight, root fresh weight and dry weight were 35%, 23%, 44%, 34%, respectively. In XLY900, the maximum decreases of the above parameters were 31%, 22%, 41%, 28%, respectively. This indicated that adversity stress severely restricted the growth and development of rice. Moreover, the inhibition effect on the biomass of HHZ was stronger than that of XLY900. Further, chlorophyll and carotenoids are necessary for plant growth and development and for the synthesis of photosynthetic products. Under salt stress, chlorophyll synthesis is prevented, which will directly affect photosynthesis, slow plant growth, and reduce yield^40,41^. In this experiment, during NaCl stress, the chlorophyll a, chlorophyll b, carotenoids, and total chlorophyll contents of the two rice varieties were significantly decreased (Fig. 5). This may be due to the increase of Na^+^ and ROS under salt stress, which disrupts the stability of the chloroplast membranes and decomposes the protein-pigment-lipid complex^42^. On the other hand, chlorophyllase enzyme activity increased to accelerate the degradation process^43^. Foliar spraying of Hemin facilitated the increase in the photosynthetic pigment content. The inhibitor ZnPP did not increase the chlorophyll content. When combined with Hemin, it reversed the downward trend and promoted the synthesis of some chlorophyll (Fig. 5A–H). The experimental results in this study are consistent with the findings of hemin mitigation efforts on *Cassia obtusifolia L*^44^. Based on the results of previous studies, it was hypothesized that Hemin increased the activity of HO-1, which had a protective effect on the photosensitive pigment chromophore. In addition, Hemin also could regulate the activity of related enzymes and promote the synthesis of chlorophyll^45,46^. In contrast, ZnPP acted as a specific inhibitor of HO-1, inhibiting the enzyme activity and to some extent reducing Hemin promotion and decreasing chlorophyll content.

Salt stress inhibited *P*_*n*_ through stomatal or non-stomatal limiting factors. If *C*_*i*_ and *G*_*s*_ decrease at the same time, this indicates that the decrease in *P*_*n*_ may be due to stomatal limitations; otherwise, it indicates non-stomatal limitations^47^. This study showed that the *P*_*n*_, *C*_*i*_, and *G*_*s*_ of the leaves in two rice varieties declined under NaCl stress (Fig. 6A–H), indicating that it was mainly stomatal limiting factors that caused the reduction in *P*_*n*_. The experimental results are consistent with findings observed in peanut^48^, cotton^49^ and mung bean^50^ and so on. Furthermore, photosynthetic pigments in chloroplasts are closely related to photosynthesis, and the decrease of pigment content in stressful environments also adversely affects gas exchange parameters^51^. Similarly, in this study, under salt stress, the chloroplast structures are disrupted in rice leaves or the plant's photosensitive mechanisms are disordered in response to stress, which may lead to a decrease in light uptake, inhibition of photosynthesis and a decrease in net photosynthetic rate^52,53^. Regardless of the cause, salt stress seriously interferes with normal plant photosynthesis, accelerates the senescence of seedlings and ultimately reduces biomass accumulation. In the present study, salt stress decreased gas exchange parameters, lowered photosynthetic pigment content and diminished biomass accumulation, which was consistent with the study of Alharbi^54^. In comparison to XLY900, HHZ accounted for a greater decrease in *P*_*n*_ under salt stress (Fig. 6A,B). This finding was consistent with the results of Chen et al.^55^, who found that salt stress caused greater damage to photosynthesis in HHZ. Under salt stress, the application of Hemin promoted the increase of *P*_*n*_, *G*_*s*_, *C*_*i*_, and *T*_*r*_ in the HHZ and XLY900 with SH treatment groups (Fig. 6A–H). This indicated that rice leaves had high levels of *G*_*s*_ and *T*_*r*_. The increase in *G*_*s*_ is beneficial to CO_2_ entry into the plant for gas exchange and carbon assimilation; the increase in *T*_*r*_ enhances the plant's ability to absorb and transport water and facilitates photosynthesis. These findings corroborate with the study of Sun et al.^36^ who demonstrated that hemin treatment under Cd stress increased maize gas exchange parameters. In addition, hemin could be effective in enhancing photosynthetic pigment content in maize leaves, improving the PSII photosynthetic system and mitigating the effects of Cd stress on photosynthetic stomata or non-stomatal limiting factors, which could enhance photosynthesis^56^.

Sucrose participates in the normal growth and development of plants by providing a source of carbon. It also functions as a signal molecule, regulating the expression of certain genes and improving plant resistance to stress^51^. Therefore, it is critical to maintain the dynamic balance of sucrose biosynthesis, transportation, and distribution for plant growth^57,58^. Previous studies have shown that salt stress increased sucrose content and the activities of sucrose phosphate synthase (SPS) and sucrose synthase (SS)^21,59^. In this experiment, salt stress resulted in an increase in sucrose and soluble sugar, and a decrease in fructose content in rice leaves (Fig. 7A,B,E–H). This may be related to the increased activity of SPS, which catalysed sucrose synthesis (Fig. 8G,H), and the reduced activity of the soluble acid invertase (SAInv), basic/neutral invertase (A/N-Inv) and SS, which catalysed sucrose catabolism (Fig. 8A–F), under salt stress. In addition, the decrease in fructose content indicated that more fructose was catalysed by SPS to produce sucrose. There was a reduction in fructose production from the breakdown of sucrose by the AI, NI and SS. Therefore, salt stress facilitated sucrose accumulation and reduced fructose content. The soluble sugar content increased, likely because the increase in sucrose was greater than the decrease in fructose or other sugar substances added. These were similar to the findings of Shao et al.^60^ HHZ accumulated relatively more sucrose and soluble sugars under salt stress than XLY900 (Fig. 7E–H). This showed that varieties which were relatively sensitive to salt stress needed to accumulate more sugars to alleviate osmotic stress. For example, the soluble sugar content of the salt-sensitive variety Jinongda138 was much higher than that of the salt-tolerant variety Changbai9^60^. However, under stressful conditions, the mass of sugars accumulated in the plant would inhibit normal photosynthesis and delay plant growth^61,62^. Therefore, in this experiment, HHZ accumulated a large amount of sugars in comparison with XLY900, which alleviated osmotic stress while further inhibited photosynthesis and thus limited its growth through feedback regulation. In this experiment, the exogenous Hemin was applied to promote sucrose catabolism for fructose production by increasing AI, NI and SS activities on the one hand (Fig. 8A–F) On the other hand, it decreased SPS activity and reduced sucrose synthesis (Fig. 8G,H). Eventually, the fructose and soluble sugar contents in rice leaves were increased to alleviate the osmotic stress caused by NaCl and maintain osmotic balance. In particular, in comparison to XLY900, the soluble sugar and fructose content were much higher in the HHZ with SH treatment group. It was possible that under salt stress, Hemin was more positive in regulating the glycoconjugates of HHZ. Therefore, more osmoregulatory substances were accumulated to maintain normal growth. These results showed similarity to Wang et al.^63^, where stress-sensitive rice varieties were more susceptible to exogenous regulatory substances. However, Zhao et al.^64^ study showed that exogenous Hemin enhanced sucrose content, improved the SPS and SS activity and reduced AI and NI activity in maize leaves, which led to an accumulation of sucrose. This was not consistent with the experimental results which found reduced sucrose content and increased fructose accumulation. It is well known that carbohydrate metabolism in plants was a complex physiological process, coordinated by the source-library-flow. The accumulation of sugars was not only associated with the catabolism of substances, but also with their transport. The leaf sucrose content was reduced, probably because more sucrose was transported to the root system for mitigation by salt stress damage. In addition, Hemin's regulation of sugars in crops could also be different owing to differences in the test material, type, and time of stress. The detailed reasons for this should be the subject of future research.

Starch is an energy-storing substance in plant cells and can be disassembled into sugars (e.g. sucrose, fructose, and glucose) to provide energy^65^. It has been reported that under abiotic stress, photosynthesis is hindered and plant accelerates the degradation of starch to maintain normal growth and development. Therefore, the plant ensures an adequate source of carbon, energy and carbon metabolites under adversity stress by regulating the production and activation of starch^65,66^. In this study, α-amylase and total amylase activities increased (Fig. 9A–D), which promoted starch breakdown. And sucrose phosphorylase (SP) activity decreased (Fig. 9E,F), which indicated a decrease in the rate of starch synthesis and ultimately a reduction in starch content and an increase in sugars in rice leaves. The abovementioned results are supported by the findings of Yan et al.^67^. Hemin enhanced the α-amylase and total amylase activities and accelerated the starch breakdown of HHZ and XLY900. This finding was confirmed in Xu et al.^68^. Theoretically, an increase in amylase activity promotes starch degradation, leading to a decrease in its content. However, in the present experiment, the starch content increased significantly, which might because Hemin improved the photosynthetic rate and SP activity. The synthesis of starch was greater than the degradation, thus the accumulation of starch was enhanced. Furthermore, in this study, the inhibitor ZnPP treatment reversed the promoting effect of Hemin. A study was found that both CO and Hemin increased the α-amylase activity and up-regulated related gene expression, which alleviated the inhibitory effect of salt stress on rice seed germination^69^. It was hypothesized that Hemin was decomposed in the plant to produce CO, which corresponded to an increase in CO content and indirectly increased the amylase activity. However, ZnPP could not increase the CO content and even inhibited the degradation of Hemin by HO-1, reducing the CO content, which produced an opposite effect to that of Hemin.

## Conclusions

The results showed that decrease in chlorophyll content, impairment of photosynthesis and a significant decrease in enzyme activities related to carbohydrate metabolism were observed in seedlings of two rice varieties under salt stress. At present, starch was heavily decomposed, while the sucrose synthesis pathway was enhanced, and leaves accumulated large amounts of sucrose. Foliar sprays of hemin improved photosynthesis by increasing net photosynthetic rate; promoting AI, NI, SS, α-amylase and total amylase activities; and decreasing SPS and SP activities. Also, the degradation of starch to fructose and sucrose was accelerated, and the content of fructose and soluble sugar in leaves was increased, which alleviated the osmotic stress caused by salt stress and maintained normal plant growth. In this experiment, the hemin was more effective in regulating HHZ in comparison with XLY900. Our study also showed that a certain concentration of Hemin can be used as a high-efficiency inducer to enhance the tolerance of rice seedlings to salt stress and improve the sustainability of rice production in saline areas. Further studies are needed to investigate the molecular mechanism of Hemin-induced salt tolerance in plant.

## Materials and methods

### Plant materials and experimental design

The experiment was carried out in 2022 in the sunlight multi-span greenhouse of College of Coastal Agricultural Sciences of Guangdong Ocean University, Zhanjiang, Guangdong Province. Seeds of two rice varieties, one inbred rice ‘Huanghuazhan’ (HHZ) and the other hybrid rice ‘Xiangliangyou 900’ (XLY900) were obtained from Guangdong Tianhong Seed Company Limited, Zhanjiang. Seeds were selected to be full and whole, and then disinfected with 3% hydrogen peroxide for 15 min, rinsed with distilled water several times until thoroughly rinsed, soaked with distilled water, and germinated for 48 h under dark conditions at 30 °C. Subsequently, 65 uniformly seeds were sown in a plastic pot (with an upper diameter of 19 cm, lower diameter of 14 cm, height of 17 cm, and no holes in the bottom); each pot was filled with about 3 kg of test soil (the volume ratio of latosol to sand was 3:1).

When the rice seedlings had naturally grown to the 3 leaf/1 heart stage (about 18 days after planting), seedlings were foliar sprayed with 5 μmol·L^−1^ Hemin (provided by Shanghai Changdeduo Agricultural Technology Co., Ltd.) and 25 μmol·L^−1^ zinc protoporphyrin IX (ZnPP, used as a specific inhibitor of HO-1) alone or in combination with a small hand-held sprayer; 10 mL of liquid was added to each pot. To avoid salinity shock, NaCl solution was added to the pots in two parts. 25 mM NaCl solution was added to each pot 24 h after spray application, and the other 25 mM NaCl solution was added at 48 h to achieve the desired salt concentration (50 mM). Concentrations were maintained by measuring the soil conductivity (EC = 5.0 ± 0.5 dS·m^−1^). The two varieties were divided into five groups of 30 pots each: (1) CK: 0 mM NaCl + 0 μmol·L^−1^Hemin; (2) S: 50 mM NaCl + 0 μmol·L^−1^Hemin; (3) SH: 50 mM NaCl + 5 μmol·L^−1^Hemin; (4) SZ: 50 mM NaCl + 25 μmol·L^−1^ZnPP; and (5) SZH: 50 mM NaCl + 5 μmol·L^−1^Hemin + 25 μmol·L^−1^ZnPP. This experiment was designed according to a completely randomized block design, with three biological repeats in each treatment. Samples were taken on days 1, 3, 5, 7, and 9 of salt stress to determine plant morphology. Physiological samples were directly cut from the leaves of potted plants and stored in liquid nitrogen in a − 40 °C freezer for physiological and biochemical analysis at 1, 3, 5, 7, and 9 d after salt stress.

### Measurement of growth indicators

At the 1st, 3rd, 5th, 7th, and 9th under NaCl stress, rice seedlings of each treatment were washed with tap water, then rinsed with distilled water, blotting paper was used to absorb surface water, above- and below-ground parts were separated for the determination of morphological indicators, and a representative population of twenty seedlings was selected for each treatment. The plant height and stem diameter of each individual were measured with a ruler and vernier. The above-ground and below-ground fresh weights were measured using an electronic balance. The rice seedlings were subsequently dried at 105 °C for 30 min and dried at 80 °C to a constant weight, and the above-ground dry weight and root dry weight were determined.

Rice seedling root systems were scanned with root system scanner (Epson Perfection V800 Photo (Epson Indonesia Inc.)), and the images were analysed and calculated using WinRHIZO root analysis software (Regent Instruments, Quebec, Canada) to obtain the total root length.

### Measurement of the photosynthetic pigments

Chlorophyll a (Chl a), chlorophyll b (Chl b), carotenoids (Car), and total chlorophyll (Chl a + b) were determined using the method proposed by Kolomeichuk^70^. Fresh leaf (0.1 g) was soaked in 10 ml 95% ethanol in the dark for 24 h. The concentration of Chl a, Chl b, and Car were measured spectrophotometrically at 665, 649, and 470 nm, respectively.

1 $$ {\text{Chl a }}\left( {{\text{mg g}}^{{ - {1}}} } \right) \, = { 13}.{\text{95 A}}_{{{665}}} - { 6}.{\text{88 A}}_{{{649}}} , $$

2 $$ {\text{Chl b }}\left( {{\text{mg g}}^{{ - {1}}} } \right) \, = { 24}.{\text{96 A}}_{{{649}}} - { 7}.{\text{32 A}}_{{{665}}} , $$

3 $$ {\text{Car }}\left( {{\text{mg g}}^{{ - {1}}} } \right) \, = \, \left( {{1}000{\text{ A}}_{{{47}0}} - {2}.0{\text{5 Chl a }} - { 114}.{\text{8 Chl b}}} \right)/{245}, $$

4 $$ {\text{Total Chl }}\left( {{\text{mg g}}^{{ - {1}}} } \right) \, = {\text{ Chl a }} + {\text{ Chl b}}. $$

### Measurement of the photosynthetic parameters

At 9:00–11:30 a.m on the 1st, 3rd, 5th, 7th, and 9th day after NaCl stress, photosynthetic parameters – including the net photosynthetic rate (*P*_n_), stomatal conductance (*G*_s_), intracellular CO_2_ concentrations (*C*_i_), and transpiration rate (*T*_r_) were determined according to Sales et al.^71^by using the LI-6800 portable photosynthesis measurement system (LI-6800, LI-COR, USA). The conditions in the leaf chamber were a photosynthetically active radiation (PAR) of 1000 μmol·m^−2^·s^−1^, CO_2_ concentration of 400 μmol·mol^−1^, leaf temperature of 30.0 ℃, relative air humidity between 70 and 80%, and air velocity of 500 μmol·s^−1^.

### Measurement of the carbohydrate content

The measurement of carbohydrate content was carried out according to the method proposed by Du et al.^72^. 0.5 g of the frozen leaf sample was placed in a mortar, ground into powder with an 80% ethanol solution (v/v) and loaded into a centrifuge tube. The centrifuge tube was placed in a water bath at 80 °C for 20 min and then centrifuged at 4000 rpm for 5 min. The supernatant was collected and the volume was fixed to 25 ml. The remaining residue was extracted three times, as described above. The resulting solution was used to measure the levels of fructose, soluble sugar, and sucrose, and the precipitate was used to measure the starch content.

The determination of fructose content was measured according to previous study^73^. For fructose, 0.8 ml supernatant was mixed with 1.6 ml of 0.1% resorcinol reagent (w/v) and 0.8 ml distilled water, heated to 80 °C for 10 min then absorbance measured at 480 nm.

For the estimation of sucrose content, 0.4 mL sugar extract was boiled with 0.2 mL of 2 M NaOH and then followed by addition of 0.8 mL of 0.1% resorcinol and 2.8 mL of 30% HCl. The reaction mixture was incubated for 10 min in water bath at 80 °C. The absorbance was measured at 480 nm using a spectrophotometer as described by Du et al.^74^.

Starch content determined by reference to the method of Kuai et al.^75^. After removing ethanol by evaporation, 2 mL of distilled water was added into the samples, and then the samples were incubated at 80 °C for 15 min. Starch was mixed with 2 ml of 9.2 M HCIO_4_ and 2 ml distilled water. The mixture centrifuged at 4000 rpm for 10 min. The residue extracted two more times with 4.6 M HClO_4_ and distilled water and collected the supernatant and fixed the volume to 50 ml. 2.5 ml supernatant was mixed with 6.5 ml anthrone reagent and absorbance was measured at 620 nm.

### Measurement of the key enzyme indexes of sucrose metabolism

The frozen leaf sample was extracted in 0.1 M PBS buffer (pH 7.5) containing 5 mM MgCl_2_, 1 mM EDTA, 1 mM EDTA, 0.1% (v/v) β-mercaptoethanol, and 0.1%(v/v) Triton X-100 at 4 °C, centrifuged at 10,000 rpm and 4℃ for 15 min. The supernatant fluid was then moved to a 10 ml calibration tube. The supernatant was used to determine the activities of acid invertase (AI), neutral invertase (NI), sucrose phosphate synthase (SPS), and sucrose synthase (SS).

The AI (EC 3.2.1.25) and NI (EC 3.2.1.26) activity were determined according to the method described by Zhu et al.^76^. The assay mixture for acid invertase contained 0.2 ml of enzyme extract, 1.8 ml of 0.1 M acetic acid buffer (pH 5.5), and 1% sucrose. The reaction progressed in a 34 °C water bath for 60 min and was stopped by boiling the mixture for 5 min in a water bath. The assay for NI activity was similar to that of AI, except that the reaction was performed in a phosphate buffer (pH 7.5). The absorbance assayed at 540 nm. Activity of AI was measured as sucrose (μg)·fresh weight (g)^−1^ min^−1^ and NI was measured as sucrose (mg)·fresh weight (g)^-1^ min^−1^.

The measurements of SPS (EC 2.4.1.14) and SS (EC 2.4.1.13) were carried out according to the methods proposed by Wongmetha^77^ and Baxter^78^. SPS was assayed in a mixed solution containing 0.1 M borate buffer (pH 8.0), 15 mM MgCl_2_, 5 mM fructose-6-phosphate, 15 mM glucose-6-phosphate, 10 mM UDP-glucose, and enzyme extract. The reaction mixtures were incubated at 30℃ for 60 min. 0.2 ml of 30% KOH was added to stop the reaction, and the mixture was then heated at 100 °C for 10 min. After cooling, anthrone reagent (in H_2_SO_4_) was added, and the absorbance was measured at a wavelength of 620 nm. The SS assay was similar to the SPS assay, but it contained 0.06 M of fructose instead of fructose-6-phosphate and was devoid of glucose-6-phosphate. Activity of SS and SPS were measured as sucrose (μg)·fresh weight (g)^−1^ min^−1^.

### Measurement of the key enzyme indexes of starch metabolism

α-Amylase (EC 3.2.1.1) and total amylase extraction and activity were measured using the method described by Dai et al.^79^ One mL of the supernatant was added to 1 mL of DNS, the mixture was heated at 100 °C for 5 min, the absorption value was determined at 520 nm after cooling, and the content of maltose was calculated from the standard curve of amylase. Activity of amylase was measured as sucrose (μg)·fresh weight (g)^−1^ min^−1^.

The starch phosphorylase (SP EC 2.4.1.7) measurements were carried out according to the method by Singh et al.^80^. Frozen leaf samples were homogenized in a buffer containing 100 mM sodium succinate (pH 5.8), 10% glycerinum, 1 mM EDTA, 15 mM β-mercaptoethanol, 1 mM EDTA, and 5 mM MgCl_2_, and centrifuged at 16,000 rpm for 10 min at 4 °C. 0.1 ml of the supernatant was mixed with 0.8 ml of SDB (100 mM sodium succinate (pH 5.8), 0.1% bovine serum albumin (w/v), 10 mM β-mercaptoethanol, 0.2 mM EDTA, and 10% glycerinum) and 0.1 ml of the substrate mixture (100 mM sodium succinate (pH 5.8), 5% soluble starch (w/v), 0.1 mM glucose-1-phosphate 0.2 mM AMP). The mixture was kept for 10 min at 30 °C; then, 2.6 ml of the solution (2.6 g ammonium molybdate in 100 ml 14% [v/v] sulfuric add) and 0.4 ml solution (0.5% stannous chloride in 0.1 mM HCl) was added. The absorption value was determined at 520 nm after centrifuging at 3000 rpm for 10 min. Activity of amylase was measured as nmol of Pi liberated μg g^-1^ min^−1^ FW.

### Statistical analysis

In this experiment, there were three replications of each indicator for each treatment. The average values and standard deviations were calculated based on experimental data. SPSS 25.0 (SPSS Inc., Chicago, USA) was used for statistical analysis. Variables of replications were compared by Duncan’s multiple-range test at a 0.05 level of significance. The figures were drawn in Origin 2021.

### Ethical approval

The use of plant materials for this study are used from Guangdong Tianhong Seed Company Limited, Zhanjiang. and has obtained the permissions of research materials. All plant experiments involved in this study are conducted in accordance with relevant regulations and guidelines.

## Data Availability

The original contributions presented in this study are included in the article, further inquiries can be directed to the corresponding author. The plant material used in this study is licensed and available for use.
